# The sustainability of exercise following colorectal surgery: A qualitative study of participants in the PREPARE-ABC trial

**DOI:** 10.1177/02692155241278936

**Published:** 2024-09-10

**Authors:** J Naisby, K Baker, K Skarparis, J Murdoch, A Clark, S Stirling, D Turner, AM Swart, J Hernon, J Saxton

**Affiliations:** 1 Department of Sport, Exercise and Rehabilitation, 5995 Northumbria University, Newcastle upon Tyne, UK; 2 Department of Nursing, Midwifery and Health, 5995 Northumbria University, Newcastle upon Tyne, UK; 3 Department of Population Health Sciences, 4616 King's College London, London, UK; 4 Norwich Clinical Trials Unit, Norwich Medical School, University of East Anglia, Norwich, UK; 5 Norwich Medical School, University of East Anglia, Norwich, UK; 6 6107 Norfolk and Norwich University Hospitals NHS Foundation Trust, Norwich, UK; 7 School of Sport, Exercise and Rehabilitation Sciences, 4019 University of Hull, Hull, UK

**Keywords:** Exercise, physical activity, cancer, qualitative study

## Abstract

**Objective:**

This study aimed to explore perceptions regarding the sustainability of exercise following participation in a pre- and post-colorectal surgery exercise intervention trial (PREPARE-ABC).

**Design:**

Qualitative interview study. Data were analysed using framework analysis and independently coded by two researchers.

**Setting:**

Six United Kingdom National Health Service Trusts.

**Participants:**

Eighteen interviews (hospital-based exercise *n* = 9, home-based exercise *n* = 3, standard care *n* = 6) were conducted with patients 12–15 months after being randomised in the trial, after their 12 month appointment.

**Intervention:**

Individuals who participated in one of two exercise intervention groups (hospital-supervised or home-supported exercise) or a standard care control group of the PREPARE-ABC trial were invited to interview.

**Results:**

The exercise interventions were reported to influence participants’ recovery and future sustainability of exercise behaviour change. Several participants continued to engage in exercise over a year after their surgery. Reasons for this included being engaged with exercise prior to diagnosis, psychological benefits of exercise and wanting to be engaged with something to help recovery. Perceptions about the sustainability of active lifestyles were influenced by confidence to engage in structured exercise or physical activity and beliefs about its potential to promote future wellness.

**Conclusions:**

Sustainability varies among individuals and early assessment of physical activity engagement could be beneficial. Physical activity interventions immediately following surgery may be important for future engagement.

## Introduction

Colorectal cancer (CRC) can have wide-ranging adverse physical and psychological health impacts.^1^ The best-proven treatment is surgical resection, with approximately 25,000 patients in the United Kingdom undergoing a major abdominal resection each year,^2^ but this can negatively affect quality of life.^3^ Pre-operative exercise that improves cardiopulmonary fitness may reduce post-operative complications^4^ and this provides the rationale for offering supported exercise programmes to people receiving treatment for cancer in the pre- and post-operative periods.^5,6^ Aside from the physiological benefits, exercise can also allow individuals to take back some control of their healthcare before, during and after cancer treatment.^7^

PREPARE-ABC is a pragmatic multicentre randomised controlled trial, designed to assess the clinical and cost-effectiveness of pre- and post-operative exercise in relation to short- and longer-term postoperative recovery outcomes in CRC patients undergoing surgical resection.^8,9^ The aim is to engage patients in exercise early in their cancer treatment journey (i.e. pre-surgical phase) and provide continued exercise support over the next 12 months as they recover from their treatment. Patients are randomly assigned (1:1:1) to hospital-supervised exercise, home-supported exercise or treatment as usual and the primary outcomes are 30-day morbidity (Clavien-Dindo) and 12-month health-related quality of life (Medical Outcomes Study Health Questionnaire). The impact of providing pre- and post-operative exercise support on longer-term exercise behaviour change in CRC patients is unknown but warrants further exploration, given evidence that habitual physical activity in the post-treatment period improves survival outcomes.^10^

A recent systematic review found that only one out of 37 included studies evaluated the long-term sustainability of exercise behaviour change amongst cancer patients offered exercise programmes during and after treatment, with the authors calling for more research into how exercise interventions can be sustained over time.^11^ CRC can impact people both physically and psychologically, with considerable variability between individuals.^12^ Thus, an improved understanding of individual and contextual barriers and facilitators to structured exercise and physical activity over the longer term in patients that have undergone treatment for cancer could help in the design of interventions for supporting long-term engagement. The aim of this study, therefore, was to explore patient perceptions regarding the sustainability of exercise behaviour change and contributing factors following participation in the PREPARE-ABC multi-centre exercise prehabilitation-rehabilitation trial.^8^

## Methods

A qualitative interview study, exploring sustainability of exercise following participation in the PREPARE-ABC trial. PREPARE-ABC was a multi-centred, three-armed, randomised controlled trial investigating the effect of hospital-supervised and home-supported exercise on short and longer-term recovery outcomes in CRC patients undergoing major lower-gastrointestinal surgery. The intervention period lasted for 12 months post-randomisation. The trial included an internal pilot phase to allow an assessment of stop/go criteria, before progression to a full trial. The PREPARE-ABC protocol and results of the internal pilot study have been reported elsewhere.^8,9^

The hospital-supervised arm involved attending up to three aerobic interval exercise sessions per week on a cycle ergometer over the 3–4 weeks prior to surgery, and two home-based resistance exercise sessions per week using resistance bands. Six weeks after surgery, patients in the hospital-supervised arm were offered monthly supervised exercise sessions at their treating hospital. Participants randomised to the home supported arm were asked to engage with current public health physical activity guidelines^13^ and also received monthly 15 min telephone counselling calls to support continued engagement in their programme. In the post-operative phase, both groups were encouraged to comply with current public health physical activity guidelines.^13^ Participants randomised to the control arm received standard care, which included no formalised pre- or post-operative support for engaging in exercise. Participants in both exercise groups received this exercise support over a 12 month period.

Process evaluation can provide critical insight for explaining the findings of randomised controlled trials, offering suggestions for improving trial implementation, and informing implications for delivering the intervention in practice and future research directions.^14^ The first stage of the mixed methods process evaluation has previously been published^15^ and included an assessment of fidelity, acceptability, and implementation issues. However, an important additional consideration is the extent to which participation in the PREPARE-ABC trial provided the motivation for continued engagement in exercise post-discharge. This study presents the results from follow-up interviews with participants at least 12 months after being randomised as a means of providing further insight into long-term exercise behaviour change. Participants randomised to the standard care control group were also included in this qualitative study to allow an in-depth exploration and comparison of longer-term exercise perceptions and behaviours between trial participants receiving and not receiving exercise support within the treatment pathway.

Participants consenting to the main trial were invited to participate in the follow-up interviews by a research nurse. It was explained that participation may involve being observed and/or interviewed by a researcher. They were provided with additional written information, informed that participation was optional, and their informed consent was secured. Participating participants provided written consent to the process evaluation, including future interviews after consenting to the main trial. However, some eligible participants were not approached about the process evaluation at the time of consenting to the main trial. In these cases, a research nurse offered participants to take part in the interview at their final 12-month appointment and informed consent was taken at this point. The study aimed to include experiences from individuals from each of the three groups. The research nurse provided information to consenting participants at 12 months. These consenting participants were then contacted by a process evaluation researcher (JN or KB) to discuss the study further and to arrange a face-to-face or telephone interview. Eighteen participants were consented and subsequently recruited to this qualitative study and their characteristics are presented in Table 1. The study was performed in accordance with the principles of the Declaration of Helsinki. The East of England – Essex Research Ethics Committee (Reference: 16/EE/0190) approved the trial at all participating sites. Consent for the process evaluation was requested separately and after participants had consented to the main trial, except for those participating in the evaluation of standard care prior to main trial recruitment.

**Table 1.** Participant characteristics.

Participant (P)	Age	Gender	Surgery/chemotherapy	Group
1	64	Male	Surgery. Chemotherapy	Standard care
2	74	Male	Surgery. Declined chemotherapy	Standard care
3	66	Male	Surgery	Home-based exercise
4	73	Male	Surgery	Hospital-based exercise
5	81	Male	Surgery. Chemotherapy	Hospital-based exercise
6	62	Male	Surgery	Home-based exercise
7	56	Female	Surgery	Hospital-based exercise
8	70	Male	Surgery. Chemotherapy	Hospital-based exercise
9	74	Male	Surgery	Hospital-based exercise
10	60	Male	Surgery. Chemotherapy	Hospital-based exercise
11	60	Male	Surgery	Home-based exercise
12	76	Male	Surgery.	Standard care
13	62	Female	Surgery. Chemotherapy	Standard care
14	51	Female	Surgery. Chemotherapy	Standard care
15	58	Male	Surgery	Hospital-based exercise
16	66	Male	Surgery	Standard care
17	80	Male	Surgery	Hospital-based intervention
18	64	Male	Surgery	Hospital-based intervention

Interviews were semi-structured around a topic guide designed to explore participant's experience of participating in the trial, their experiences of being diagnosed with and receiving treatment for bowel cancer, previous habits and motivation to exercise, changes following participation in the study and perceived links between being physically active and their trajectory of recovery. The concept of recovery was directed by participants as to what was important to them for example physical fitness and quality of life. Consenting participants were contacted by a process evaluation researcher (JN or KB) to discuss the study further and to arrange a face-to-face or telephone interview. A purposeful sample capturing each of the trial arms, from different National Health Service (NHS) sites was conducted. Individuals coming up to their 12 month appointment who had previously consented to be involved in interviews for PREPARE-ABC or had not been approached about interviews were invited. We aimed to recruit individuals from different sites and different arms of the study. We communicated demographics of the sample to the NHS sites and the aims of the purposeful sample. Recruitment was led by the research staff at the NHS sites, not PREPARE-ABC staff, thereby providing distance from the recruitment. All interviews were conducted by telephone, except for one (P3), which was face to face. JN conducted 14 interviews and KB conducted four. On average interviews lasted 25 min (14.07–42.27). JN and KB are academic physiotherapists, however, were not involved in the delivery of PREPARE-ABC trial.

The 18 interviews were digitally recorded and transcribed verbatim by an independent transcriber. Principles of thematic analysis were used both in the development of the thematic framework and in the analysis of the interview transcripts, with a framework approach being used to manage the data.^16,17^ Two authors (JN and KB) independently coded the first three transcripts to develop a coding framework. The researchers split the remaining 15 transcripts, applying the thematic framework to these. The researchers met to discuss the developed thematic framework, refined, and finalised this and applied any new findings to their transcripts. This process ensured that different perspectives were considered and contributed to the overall refinement of results. Following development of the final thematic framework, data were summarised into a chart for each theme, sub-theme and participant. This charting stage involved examining the data to explore similarities and contrasts and supported refinement of the overall themes.^17^ This approach provided a clear audit trail of the data analysis process.^18^ The 18 interviews provided sufficient information power capturing experiences of hospital, home and standard care, and understanding those with different physical activity backgrounds.

## Results

Participants were recruited from six different NHS sites participating in the PREPARE-ABC trial. Nine participants had been randomised to the hospital-supervised exercise intervention, three to the home-supported exercise intervention and six to the standard care control group. Two main themes emerged from the thematic analysis: ‘Early engagement in physical activity influences recovery and future sustainability’ and ‘PREPARE-ABC as a foundation for sustainable physical activity behaviour change’.

### Early engagement in physical activity influences recovery and future sustainability

#### An active lifestyle prior to surgery and during recovery

Many participants reported being physically active prior to enrolment onto the PREPARE-ABC trial. Individuals participated in a range of activities including walking, cycling and the gym. Through this previous experience, a common thread that emerged was an awareness of the benefits of exercise, and the impact this has on overall health and wellbeing. Participants with previous experience from each of the three groups understood the importance of keeping physically active.

*P2: When I retired from work, I decided I wasn’t going to sit on my backside all the time and keep myself active … Which I did. And I’m still doing.*

*P3: Well, it is generally anything that gets the blood circulating and the lungs going, you know. And it doesn’t matter whether you’re walking, swimming, riding a bike or flogging your guts out in a sweaty gym, you know. It's all the same … The main thing is just to stay active.*

There were some participants who did not view themselves as active prior to diagnosis. This varied from not ‘counting’ activities such as regular walking as physical activity, to associating only going to the gym with being physically active. However, discussions revealed that several individuals dedicated a lot of their time to walking or physically active hobbies. A small number of participants said physical activity was not a key part of their lives prior to diagnosis, but that the diagnosis of cancer prompted them to consider incorporating this into their daily routines. This new outlook was linked with the ‘turning point’ of diagnosis encouraging a greater focus on physical activity and wellbeing.

*P07 When he asked me, you know, “Do you want to have a go and sign up for this research programme – it's fitness…” You know … How fitness effects bowel cancer … There was no way I would ever have thought not to sign up for it.*

The majority of individuals placed priority on becoming physically active soon after surgery. This was influenced by them not wanting to remain in bed and to be doing something which would positively impact recuperation. Participants were often surprised that they were up and walking around quickly after surgery. Across each of the three groups, early mobilisation was discussed and encouraged by healthcare professionals as standard practice. Being back to ‘normal’ as soon as possible was a key priority for participants and physical activity was a means to achieve this.

*P16: But I think anybody who has major surgery, really, has to take the clinician's advice and actually not do too little. You really have to make sure you’re moving about, you know.*

*P3: Well, you know, the object of the exercise after the op was to get back to normal. So that was the ultimate goal, to be back as close to normal as possible. And in that respect, yes, I’m being fairly successful on that.*

Once discharged from hospital, most participants in each of the groups discussed a gradual increase in physical activity. Once individuals felt comfortable being able to walk increased distances, some then progressed onto structured exercise programmes (e.g. going to the gym) or physical activity as an integral part of hobbies. Seeing progress made in the distance walked during the weeks after surgery motivated participants to keep going.

*P11: three months previous, when I came out of hospital … you were looking at hundreds of metres as being the absolute limit. But I walked … You know, we walked this six miles and absolutely no … You know, didn’t even feel like I’d done it, sort of thing.*

*P14: at first, it was probably just walks around the estate. So … For quite a while I couldn’t walk the dog, just in case he pulled. So, my neighbours were walking him for me. But then … Then I would walk round with them, but just not holding the dog. And then I just gradually built myself back up.*

Six participants started chemotherapy following their surgery (three in the hospital-supervised exercise group and three in the standard care control group). Tiredness was a prominent side effect that influenced participants’ ability to engage in physical activity. Participants could clearly recall the unpleasantness of chemotherapy and provided detail regarding their experiences during treatment and the impact this had post-surgery and into recovery. Each of these participants, regardless of group allocation, focused on becoming more active immediately post-surgery, in accordance with current standard care guidance. However, a member of the standard care group said they would have liked to have received further support and guidance on exercise at this stage.

*P8 I didn’t realise how hard that was. You know, I walked up and down for maybe five/six minutes. I was absolutely knackered to begin with.*

*P14: You know, exercises … And the one thing I wish they’d sort of send me away with was like a list of exercises, after the operation.*

#### The perceived role of exercise post-operatively

Participation in exercise post-surgery was viewed as helping with recovery. As this had been discussed with the surgeon or other healthcare professional as part of the trial, participants trusted the potential health benefits of exercise in this context. For individuals who were physically active and those who were sedentary prior to diagnosis, this was a key theme. Recovery and exercise were viewed as being closely linked and individuals were choosing to exercise as means of enhancing their recovery.

*P3: And, you know, it just went so well. And because it went so well, probably assisted the recovery time.*

*P4: Feel it can only have done me good to… You know, to do the exer… Well, to be involved in the programme and to have done the extra exercises I’ve been doing.*

The psychological impact of engaging in exercise was reflected upon by some participants. Feeling good in themselves and an overall sense of wellbeing was captured through participation in the exercise programmes. Furthermore, participants felt they were ‘doing something’ positive to contribute to their recovery. Those participating in regular exercise or purposeful physical activity discussed how they hoped this would reduce the likelihood of their cancer returning or at least ensure that they were as fit as possible if this were the case.

*P2: Well it gives me a better sense of wellbeing. Feeling that I was actually doing something to keep healthy.*

*P7: You know, I am far more motivated than I have ever been to get back to better than I was before I was ill.*

*P15: One of the benefits of the exercise is that you just get a sort of feel of wellbeing, just doing those things, rather than stagnating. So it's … It is a … It feels there's a mental benefit of doing those things rather than sitting around and so on.*

### PREPARE-ABC as a foundation for sustainable physical activity behaviour change

#### Support from PREPARE-ABC to maintain exercise behaviour change

The hospital-supervised and home-supported exercise participants expressed praise for the continued support they had received up to 12 months post-surgery during their participation in PREPARE-ABC. Participants provided detailed recollections of the motivation provided by healthcare professionals and the confidence that this gave them to engage in exercise and become more active. Participants who were both active and inactive prior to diagnosis found PREPARE-ABC to be informative and reassuring, regarding the exercise support received.

*P17: I wouldn’t have recovered like I did without [the exercise] – prior to going into hospital and also when I’d finished, you know, the … The physio was the bit that got me back onto full health, really.*

The home-supported exercise group viewed the flexibility of home exercise in the 12 months post-surgery very positively, including their capability to develop their exercise programme in this environment. Telephone calls from staff members provided the motivation needed for filling in activity diaries.

*P3: I was one of the lucky ones that didn’t have to go in for supervised exercise or gym visits and all the rest of it. And it was just … Basically I increased my activity at home.*

#### Strategies for maintaining an active lifestyle

Participants who participated in the hospital-supervised or home-supported exercise group demonstrated variation in the overall impact of participation in PREPARE-ABC on their current physical activity levels. Most participants maintained some form of physical activity beyond the trial, though there was variation in the type, frequency, and intensity of physical activity and structured exercise between participants. Influencing factors included age, other comorbidities, and time. Some participants invested in exercise bikes because of finding this type of exercise beneficial when taking part in the programme. Other individuals adapted exercises from the PREPARE-ABC programme to fit into their daily routine. They described how PREPARE-ABC provided some initial structure to their exercise routine, that over the course of their recovery they could adapt and fit into their daily lives.

*P7: I think it's been brilliant. I think I would not be where I am now if it hadn’t have been … If I hadn’t have signed up for this and I hadn’t had that physio and she’d led me down the path she's led me. You know, still, a lot of the stuff she's done with me, I am still doing now. You know, I had a couple of months off my cross-trainer in the winter after I’d finished competing, while I was doing other things.*

*P15: followed the guidance from the surgery team and then when that … That finished, sort of moved into more regular exercise.*

Those who were active before PREPARE-ABC often maintained either similar habits or more exercise in the longer-term, incorporating activities they enjoyed into daily life. The exercise programmes in PREPARE-ABC provided ideas and inspiration for participants in the home-supported and hospital-supervised exercise groups. Whilst they may not have maintained the specified exercises, these provided a basis for progression and individuals being able to choose what they enjoy, to maintain active lifestyles over the longer-term.

*P15: So, even though I had the rubber bands … I used that for a few days. After that I got onto, you know, rowing machine and cycling machine and jogging machines and things like that in my gym.*

*P4: I was pretty active in other things and didn’t specifically … You know, occasionally I’ll still use them [exercises from PREPARE-ABC] now and do a few stretches on the arm muscles and that sort of thing.*

Keeping fit and strong became a priority for previously active participants that were allocated to the home-supported and hospital-supervised exercise groups. For them, PREPARE-ABC had further confirmed their beliefs about the importance of physical activity and encouraged longer-term participation in active lifestyles. This was bolstered by their newly developed knowledge about the safety aspects of exercise, its key role in recovery and the importance of an active lifestyle for improving their chances of returning to pre-diagnosis levels of activity.

*P18: I’ve doubled my … I’ve at least doubled my exercise regime. Mainly because, like, if it does happen again, I want to be in the best possible place.*

Two individuals in the hospital group reported being sedentary prior to participating in PREPARE-ABC. Physical activity had not previously been an important part of their lives. For these participants, PREPARE-ABC had provided the motivation to engage in physical activity and the confidence to maintain an active lifestyle. Whilst the individuals were aware of the health benefits of exercise prior to their diagnosis, their involvement in PREPARE-ABC and subsequent engagement in exercise provided them with a new focus and increased the priority of physical activity.

*P17: Probably wasn’t getting the exercise that I should get, especially as I’m a petrol-head, so I go everywhere by car.*

*P10: I suppose the diagnosis gave me a focus to really put some effort in. But I … You know … I’ve been (a member of) the gym for quite a while. It's just sometimes I didn’t get round to it.*

Importantly, participation in PREPARE-ABC enabled these individuals to try exercise in a supported way. Enjoyment was a key term highlighted by the individuals who were mainly sedentary prior to diagnosis. Being engaged in frequent exercise and developing a sense that exercise was something they wanted to do, encouraged longer-term continuation of exercise participation throughout the 12 month follow-up period. This enjoyment and ‘feel-good factor’ also prompted these individuals to reflect during periods when exercise habits declined, leading them to want to get back to the positive feelings exercise participation provided.

*P17: And now, at this point in time, I feel absolutely wonderful, apart from the fact that I can’t walk properly. But, in myself, I feel great. And it's all down to … It's all down to the exercise.*

For those both active and sedentary individuals, prior to PREPARE-ABC, finding enjoyment in exercise and what suited the individual and their needs was an important consideration for longer-term sustainability. PREPARE-ABC provided this foundation, whether this was introducing individuals to exercise or providing exercise at an individualised level that suited those already physically active. The support from PREPARE-ABC and flexibility of exercise options in the follow-up period suited different physical capability levels.

Some participants who were not allocated to an exercise group were pleased to be allocated to no intervention so that they would not have to attend appointments and exercise within a hospital gym. Participants in this group expressed wanting to join the trial to ‘give something back’ and support future research. Just being part of the trial provided a platform for the standard care participants to reflect on their health. There was evidence that even being part of the standard care only arm of the trial, impacted physical activity behaviours. For some of this group, longer-term engagement in exercise was centred around walking.

*P2: Well, the curiosity that you people have had towards me. To me it's sort of helped with my general outlook and stuff like that, you know.*

*P14: He mentioned it then and he said, you know, “Would you be interested?” And I went “Absolutely! If I can help, in any way, shape or form, on the … You know, the journey I’m on, or I’ve got to go on, then, by all means, I will”.*

*P13 I don’t do any, like, strenuous exercise like cycling or weights … It's just been purely walking and sort of general, fairly light housework, you know.*

## Discussion

The aim of this study was to explore factors influencing the sustainability of exercise in CRC participants following participation in a pre- and post-operative exercise intervention trial (PREPARE-ABC). Including both intervention and standard care participants enabled an inclusive exploration of sustainability and to explore the contribution of PREPARE-ABC. Participants who were mostly inactive prior to diagnosis and enrolment onto PREPARE-ABC, found that involvement in the trial motivated them to become more active and maintain a physically active lifestyle over the 12-month follow-up. Some other participants engaged in regular higher intensity exercise, including strengthening exercises. This highlights the applicability of PREPARE-ABC, being suited to a range of CRC patients with different prior experiences of physical activity.

Our findings showed that a diagnosis of CRC was a ‘turning point’ for many, in which behaviour change interventions such as exercise can be of interest in the context of wanting to make positive change to optimise post-operative health. This has been described in the literature as a ‘window of opportunity’^19^ or ‘teachable moment’^20^ in which participants are more receptive to health behaviour change. PREPARE-ABC aimed to prepare patients for surgery, as well as being an integral part of the recovery process, by facilitating engagement in physical activity. Consistent with previous research exploring exercise for people undergoing surgery for cancer, our results suggest that participants liked the idea of helping themselves and being part of the recovery process.^21^ It gave participants a sense of control. The interrelated nature of the initial structured intervention, the feeling of some control and sustaining physical activity was evident here. Importantly, exercise may not be consistently advocated within the cancer treatment pathway and the ‘teachable moment’ opportunity can be missed.^22^

Notably, exercise perceptions differed between participants. For example, some participants did not consider walking as physical activity, and this is something to consider in future as a means of motivating certain participants for long-term exercise behaviour change. It is helpful for patients to understand that a physically active lifestyle does not necessarily mean going to a gym and it must suit the individual's environment and identity.^21^ Although this is reflected in current public health guidance, recent research shows that many health professionals are unaware of this or are not promoting current guidance.^23^ Interestingly, walking as a means of remaining engaged in active lifestyles over the long-term dominated discussions, particular with standard care group participants. Whereas in comparison to the intervention groups, strengthening exercise was not as frequently discussed. In accordance with the Chief Medical Officer Physical Activity Guidelines,^13^ muscle strengthening exercises should also be considered. The intervention groups discussed how PREPARE-ABC had provided ideas for muscle strengthening, including resistance bands and gym activities, and these were continued into the future.

Our results provide evidence from individuals’ experiences that receiving pre- and post-operative exercise support can facilitate sustainable exercise behaviour change over 12 months of follow-up. Furthermore, participant perceptions suggest that early encouragement to exercise could be influential in the motivation to sustain physical activity behaviour change over a 12 month follow-up period. This extends previous research which shows the health benefits of exercise during and after CRC treatment^6^ but which has not explored patient perceptions of how supported participation in early-phases of treatment influences motivation for longer-term behaviour change. Our findings extend to participants who had undergone adjuvant treatment. Patients undergoing chemotherapy find exercise very challenging and are likely to regard it as low priority if it is not deemed important for optimising recovery.^24^ However, whilst barriers such as severe fatigue were recounted by these patients, such barriers did not stop them from engaging in exercise or maintaining a physically active lifestyle in the 12 month follow-up period. Thus, it seems that the advice and support received via PREPARE-ABC helped participants to overcome key barriers and concerns associated with adjuvant treatment. For many participants, engaging in exercise around the time of treatment appeared to build psychological resilience and a mindset that enabled them to remain motivated over the 12 month follow-up period. Within behaviour change literature, early promise in sustainability has been found with those who create a new identity as a physically active person.^25^

Many participants were physically active prior to taking part in PREPARE-ABC which may influence the transferability of the results due to these individuals being more interested in physical activity. However, light physical activity dominated the trial sample.^9^ Future studies should aim to capture views of more inactive participants who are recruited to exercise studies to uncover barriers and facilitators to long-term participation amongst those with less motivation for physical activity. A limitation is that this study did not recruit those with low reported adherence to the intervention. However, this qualitative study demonstrates several strengths. Data analysis was completed by two independent researchers, enhancing the credibility of the research. Framework analysis provides a clear audit trail of the analysis process and enhances dependability of the study findings. The study was able to recruit a range of ages, gender, NHS location, treatment, and group allocation, enhancing the transferability of findings. The study team encompasses a multidisciplinary range of healthcare professionals, exercise scientists and methodological experts. This has enhanced the quality of the presented data analysis, gaining multiple perspectives on the themes generated.

Within this study, continued engagement in physical activity was reported by many cancer survivors up to and over a year since surgery for CRC. There is evidence that support provided by PREPARE-ABC motivated participants to remain active over 12 months. If exercise was a priority early in the care pathway, this often was maintained into the future. Therefore, early intervention from recovery, for example through rehabilitation could enhance motivation for long term exercise behaviour change. However, the longer term impact of early rehabilitation requires further research. There were some individuals who were inactive prior to enrolment on PREPARE-ABC who reported sustaining physical activity in the longer term. For these people it was the role of physical activity and wellness, creating a new identity that involved being physically active and holding self to account that enabled this behaviour. Future research is required to understand the impact of varying levels of prior physical activity on longer term treatment outcomes for patients undergoing surgery for CRC. Future studies should aim to capture views of more inactive patients who are recruited to exercise studies to uncover barriers and facilitators to long-term participation amongst those with less motivation for physical activity.

**Clinical messages**

- Supported exercise programmes, around the time of surgical treatment and beyond can provide the foundation for sustainable exercise behaviour change amongst CRC patients.
- Exercise early in recovery could enhance motivation for longer term engagement.
